# Effects of fermented potato protein supplementation on serum insulin-like growth factor-1 (IGF-1) concentrations, skeletal muscle IGF-1 mRNA expression, and the interval to first postpartum estrus in tropical crossbred dairy cows

**DOI:** 10.14202/vetworld.2026.3608-3620

**Published:** 2026-08-17

**Authors:** Somchai Sajapitak, Sittipoj Krasaesub, Pongpak Intaravichai, Supot Awsakulsuddhi, Techaphon Songphasuk, Narut Thanantong

**Affiliations:** 1 Department of Large Animal and Wildlife Clinical Sciences, Faculty of Veterinary Medicine, Kasetsart University, Kamphaeng Saen, Nakhon Pathom 73140, Thailand.; 2 Bureau of Veterinary Biologics, Department of Livestock Development, Nakhon Ratchasima, Thailand.; 3 Division of Veterinary Inspection and Quarantine, Department of Livestock Development, Mueang Pathum Thani, Pathum Thani 12000, Thailand.; 4 Department of Farm Resources and Production Medicine, Faculty of Veterinary Medicine, Kasetsart University, Kamphaeng Saen, Nakhon Pathom 73140, Thailand.

**Keywords:** agro-industrial by-product, dairy cattle, endocrine regulation, fermented potato protein, insulin-like growth factor-1, postpartum estrus, reproductive performance, somatotropic axis

## Abstract

**Background and Aim:**

Metabolic adaptation during the transition period is a major determinant of reproductive performance in dairy cows. Insulin-like growth factor-1 (IGF-1), a key regulator of the somatotropic axis, links nutritional status with endocrine function and postpartum reproductive recovery. Fermented potato protein (FPP), a value-added agro-industrial by-product, has improved protein digestibility and may contain bioactive compounds that modulate metabolic pathways. However, its effects on endocrine regulation and reproductive performance in tropical crossbred dairy cattle remain poorly understood. This study aimed to evaluate the effects of FPP supplementation on serum IGF-1 concentrations, skeletal muscle *IGF-1* mRNA expression, and the interval from calving to first postpartum estrus.

**Materials and Methods:**

Two experiments were conducted. In Experiment 1, ten clinically healthy *Bos taurus* × *Bos indicus* crossbred dairy heifers were randomly assigned to either a control group or an FPP-supplemented group (30 g/head/day; n = 5/group) for 45 days. Serum IGF-1 concentrations were quantified using enzyme-linked immunosorbent assay, whereas skeletal muscle *IGF-1* mRNA expression was determined by quantitative real-time polymerase chain reaction. In Experiment 2, twenty-four transition dairy cows (parity 1-2; n = 12/group) received either the basal diet or FPP supplementation from 3 weeks prepartum to 6 weeks postpartum. The interval from calving to first postpartum estrus was monitored using an automated pedometer and confirmed by transrectal ultrasonography.

**Results:**

FPP supplementation significantly increased serum IGF-1 concentrations on day 30 (17.97 ± 4.93 vs. 7.82 ± 0.87 ng/mL; p = 0.016) and day 45 (18.45 ± 4.13 vs. 7.72 ± 0.75 ng/mL; p = 0.016), whereas no significant differences were observed on days 0 and 15 (p > 0.05). Skeletal muscle *IGF-1* mRNA expression did not differ between groups (0.82 ± 0.07 vs. 0.84 ± 0.14; p = 0.90). Cows supplemented with FPP exhibited a numerically shorter interval from calving to first postpartum estrus than controls (43.83 ± 2.40 vs. 50.00 ± 2.06 days), although the difference was not statistically significant (p = 0.06).

**Conclusion:**

FPP supplementation increased circulating IGF-1 concentrations without altering skeletal muscle *IGF-1* mRNA expression, suggesting that its biological effects are mediated primarily by systemic endocrine regulation rather than by local tissue-specific mechanisms. Although improvement in postpartum reproductive recovery was not statistically significant, the concurrent increase in circulating IGF-1 and shorter estrus interval indicate that FPP may represent a sustainable nutritional strategy for improving metabolic adaptation in tropical dairy cattle. Larger studies are warranted to confirm its reproductive benefits and establish its role in precision nutritional management.

## INTRODUCTION

Reproductive efficiency is one of the most important determinants of productivity and economic sustainability in modern dairy production systems. Despite substantial genetic progress in milk production, reproductive performance in high-producing dairy cows has declined in many commercial herds worldwide, largely because of metabolic and physiological challenges associated with intensive lactation [1–6]. Prolonged intervals from calving to first ovulation, delayed estrus expression, and reduced conception rates are frequently observed during the early postpartum period and represent major constraints to herd fertility and profitability [2, 5, 6]. Tropical dairy production systems face additional challenges, including chronic heat stress and seasonal fluctuations in forage availability, which impair metabolic adaptation and delay postpartum reproductive recovery. In Southeast Asia, the postpartum anestrous interval often exceeds 50 days, leading to prolonged calving intervals and increased production costs [7–9]. Consequently, nutritional interventions that improve metabolic adaptation during the transition period are essential, as even modest reductions in the interval to first postpartum estrus can substantially improve reproductive efficiency, economic returns, and lifetime productivity in smallholder dairy systems [3, 4, 10, 11]. These reproductive disturbances are primarily driven by complex interactions among metabolic status, endocrine regulation, and nutrient partitioning during the transition from late gestation to early lactation [4, 10, 11].

Reproductive function in cattle is regulated principally through the hypothalamic–pituitary–gonadal (HPG) axis. Pulsatile secretion of gonadotropin-releasing hormone (GnRH) from the hypothalamus stimulates the anterior pituitary gland to release luteinizing hormone (LH) and follicle-stimulating hormone (FSH), which subsequently regulate follicular development, steroidogenesis, and ovulation [12–14]. However, during early lactation, high-producing dairy cows commonly experience negative energy balance (NEB) because energy expenditure for milk production exceeds dietary energy intake [3, 15]. This metabolic challenge disrupts endocrine signaling within the HPG axis by suppressing hypothalamic GnRH secretion and reducing gonadotropin release, thereby delaying ovarian cyclicity and impairing reproductive performance [13, 15].

Among the metabolic mediators linking nutritional status with reproductive physiology, insulin-like growth factor-1 (IGF-1) plays a central role. IGF-1 is synthesized predominantly in the liver in response to growth hormone stimulation and represents a key component of the somatotropic axis, which integrates nutrient availability with metabolic and reproductive functions [16, 17]. In addition to hepatic synthesis, IGF-1 is expressed in several peripheral tissues, including skeletal muscle, adipose tissue, and ovarian follicles, where it exerts endocrine, paracrine, and autocrine effects that regulate cellular proliferation, differentiation, and metabolic homeostasis [18–20]. Through these mechanisms, the somatotropic axis coordinates nutrient partitioning among growth, lactation, and reproduction in dairy cattle [10, 17, 21, 22].

Substantial evidence demonstrates that circulating IGF-1 is closely associated with ovarian function and fertility in dairy cows. Increased circulating IGF-1 concentrations promote follicular growth, stimulate granulosa cell proliferation, enhance estradiol synthesis, and increase follicular responsiveness to gonadotropins, thereby facilitating follicular selection and ovulation [23–25]. Conversely, reduced circulating IGF-1 concentrations during early lactation are associated with impaired follicular development, delayed ovulation, and reduced fertility in cows experiencing metabolic stress [2, 26, 27]. Consequently, circulating IGF-1 is widely recognized as an important metabolic biomarker of reproductive competence in dairy cattle [23, 27]. Furthermore, recent field studies have demonstrated close associations among circulating IGF-1, insulin-like growth factor-binding proteins (IGFBPs), metabolic indicators such as β-hydroxybutyrate, and follicular signaling pathways, highlighting the integrative role of the somatotropic axis in coordinating metabolism and reproduction [28].

Recent advances in reproductive neuroendocrinology have further expanded understanding of the mechanisms through which metabolic signals regulate reproductive function. In particular, hypothalamic kisspeptin neurons have emerged as major regulators of GnRH secretion and are now recognized as central integrators of metabolic and reproductive signals [29–32]. Kisspeptin, encoded by the Kisspeptin 1 (*KISS1*) gene, stimulates GnRH neurons and subsequently activates the HPG axis [29, 30]. Experimental evidence suggests that IGF-1 may regulate this pathway by stimulating *KISS1* gene expression and enhancing kisspeptin neuronal activity, thereby promoting GnRH secretion and gonadotropin release [33–35]. These findings indicate that metabolic signals such as IGF-1 may regulate reproductive activity not only through direct ovarian actions but also through central neuroendocrine mechanisms.

Given the close relationship between metabolic status and reproductive performance, nutritional strategies that improve metabolic adaptation during the transition period have received increasing attention. Adequate dietary protein intake and amino acid availability are essential for maintaining metabolic homeostasis and supporting endocrine signaling pathways involved in IGF-1 synthesis [36–38]. Dietary amino acids influence hepatic metabolism and activation of the growth hormone-IGF axis, thereby affecting circulating IGF-1 concentrations and potentially improving reproductive function [36, 38, 39].

Fermented feed ingredients derived from agro-industrial by-products have recently attracted considerable interest as functional nutritional supplements for livestock. Microbial fermentation improves protein digestibility, increases the availability of essential amino acids, and generates bioactive peptides that modulate metabolic and endocrine signaling pathways [36, 38, 40]. Among these products, potato protein, a by-product of the potato starch industry, represents a promising alternative protein source for ruminants. Fermented potato protein (FPP), commercially available as Lianol® Dairy, is produced through microbial fermentation of potato-processing by-products. In addition to improving protein digestibility and amino acid availability, fermentation generates biologically active metabolites, including indole derivatives such as indole-3-acetic acid, serotonin, tryptamine, and related indole compounds [41]. These metabolites have been proposed to influence hepatic metabolism and endocrine regulation within the somatotropic axis, although direct evidence in ruminants remains limited.

Experimental studies in several animal species have demonstrated beneficial physiological effects of FPP and fermentation-derived bioactive compounds. In pigs, FPP improves growth performance, survival, metabolic status, and circulating IGF-1 concentrations. Interestingly, these increases in circulating IGF-1 are associated with increased skeletal muscle *IGF-1* mRNA expression rather than hepatic transcription, suggesting that skeletal muscle may contribute to local autocrine and paracrine regulation independently of hepatic endocrine production [42]. Similarly, fermentation-derived products containing indole-3-acetic acid have been reported to increase circulating IGF-1 concentrations in dairy cows, piglets, and laying hens, although the underlying biological mechanisms remain unclear [38, 41–43].

Although interest in fermented feed ingredients has increased substantially, important knowledge gaps remain regarding the endocrine and reproductive effects of FPP supplementation in dairy cattle. Previous studies have mainly evaluated circulating endocrine responses and production outcomes, whereas tissue-specific molecular regulation has received little attention. For example, supplementation with fermented concentrated potato protein increased circulating IGF-1 concentrations after 21-30 days and numerically improved pregnancy rate and estrus interval in Holstein cows, but these improvements were not statistically significant, and the biological mechanisms underlying these responses remained unresolved [41]. Furthermore, the available evidence has been generated primarily in temperate Holstein cattle or monogastric species, limiting its applicability to tropical crossbred (*Bos taurus* × *Bos indicus*) dairy cattle, whose metabolic adaptation, endocrine regulation, and responses to nutritional interventions differ because of chronic heat stress, variable feed quality, and distinct physiological adaptations. Consequently, whether FPP influences systemic endocrine regulation, local tissue-specific *IGF-1* expression, and postpartum reproductive recovery in tropical dairy cattle remains unknown.

To our knowledge, this is the first study to simultaneously evaluate the endocrine, molecular, and reproductive responses to FPP supplementation in tropical crossbred dairy cattle. We hypothesized that dietary FPP supplementation would increase circulating IGF-1 concentrations by modulating the somatotropic axis, and we sought to clarify whether skeletal muscle *IGF-1* expression contributes to these endocrine responses and whether these changes are associated with improved postpartum reproductive recovery. The supplementation level of 30 g/head/day was selected based on the manufacturer's recommendation and previous studies demonstrating biological responses at inclusion rates of 25-50 g/head/day [41]. This commercially relevant dosage provides adequate exposure to fermentation-derived bioactive compounds without adversely affecting feed intake or palatability. Therefore, the objective of this study was to determine the effects of dietary FPP supplementation on serum IGF-1 concentrations, skeletal muscle *IGF-1* mRNA expression, and the interval from calving to first postpartum estrus in tropical crossbred dairy cows.

## MATERIALS AND METHODS

### Ethical approval

All experimental procedures involving animals were reviewed and approved by the Institutional Animal Care and Use Committee (IACUC), Kasetsart University, Bangkok, Thailand (Approval No. ACKU04660). The study was conducted in accordance with the National Research Council of Thailand Guidelines for the Care and Use of Animals for Scientific Research and complied with internationally accepted principles for the ethical use of animals in research. The study was designed, conducted, and reported in accordance with the ARRIVE 2.0 (Animal Research: Reporting of *In Vivo* Experiments) guidelines to ensure transparent and reproducible reporting of animal experiments.

Before enrollment, all animals underwent comprehensive veterinary examinations, and only clinically healthy animals free from systemic disease, lameness, or reproductive disorders were included. Throughout the study, animals were maintained under routine veterinary supervision, including regular health assessments, vaccination, parasite control, and appropriate husbandry practices. Feed and clean drinking water were provided *ad libitum*, and animal health and welfare were monitored daily.

Blood collection was performed by experienced veterinarians using standard aseptic procedures to minimize stress and discomfort. Skeletal muscle biopsies were conducted under local infiltration anesthesia with 2% lidocaine using sterile techniques. Following tissue collection, hemostasis was achieved with gentle pressure; the biopsy site was disinfected with povidone-iodine; a topical antibiotic spray was applied; and animals were monitored daily for wound healing, appetite, locomotion, and signs of pain, inflammation, or infection. No biopsy-related complications or adverse welfare events were observed.

The principles of Replacement, Reduction, and Refinement (3Rs) were incorporated throughout the study by using the minimum number of animals necessary to achieve the scientific objectives, minimizing invasive procedures wherever possible, and ensuring that all procedures were performed by trained personnel to reduce pain, stress, and unnecessary animal suffering.

### Study period and location

The study was conducted from September 2018 to May 2019 at the dairy research facilities of Kasetsart University, Kamphaeng Saen Campus, Nakhon Pathom Province, Thailand. Both experiments were performed under standard commercial dairy management conditions with routine veterinary supervision, including vaccination, parasite control, and regular health monitoring.

### Study design

This study consisted of two independent randomized controlled experiments designed to evaluate the effects of FPP supplementation on endocrine, molecular, and reproductive responses in tropical crossbred dairy cattle. Experiment 1 evaluated changes in circulating IGF-1 concentrations and skeletal muscle *IGF-1* mRNA expression, whereas Experiment 2 investigated the effect of FPP supplementation on the interval from calving to first postpartum estrus.

### Experimental animals

Before enrollment, all animals underwent comprehensive clinical examinations, and only clinically healthy animals without evidence of systemic illness, lameness, or reproductive disorders were included. Throughout the study, animals remained under continuous veterinary supervision with routine health assessments, vaccination, parasite control, and monitoring to ensure that concurrent diseases did not influence the experimental outcomes.

### Experimental design

**Experiment 1:** Ten clinically healthy crossbred Holstein–Friesian dairy heifers (87.5%-93.75% *Bos taurus* × *Bos indicus*), aged 16-18 months and weighing 256-307 kg (mean ± Standard error of the mean [SEM]: control = 279.6 ± 6.81 kg; FPP = 279.4 ± 8.84 kg), were housed under identical management conditions and randomly allocated in a completely randomized design to either a control group receiving the basal diet or an FPP-supplemented group (30 g/head/day; n = 5/group). The basal diet consisted of concentrate and roughage formulated to meet nutrient requirements.

The FPP used in this study was a commercial feed additive (Lianol® Dairy; Huvepharma, Antwerp, Belgium), produced through controlled microbial fermentation of potato-processing by-products. The fermentation process hydrolyzes potato proteins into highly digestible protein fractions and low-molecular-weight bioactive peptides while generating essential amino acids and indole metabolites that may support hepatic metabolic function. Previous studies in monogastric species have demonstrated improvements in endocrine function, circulating IGF-1 concentrations, growth, metabolic recovery, and survival following FPP supplementation. However, these mechanisms have not been confirmed in dairy cattle. The supplementation level of 30 g/head/day was selected based on the manufacturer's recommended inclusion rate (25 g/head/day) and previous studies demonstrating biological responses at 25-50 g/head/day [41]. This dosage enabled evaluation of endocrine and reproductive responses without altering the nutritional composition of the basal diet. Animals received the experimental diets for 45 days and were fed twice daily with unrestricted access to clean drinking water.

**Experiment 2:** Twenty-four transition dairy cows (parity 1-2), aged 2.8-5.2 years and having body condition scores of 3.0-3.25 (5-point scale), were enrolled from 3 weeks before the expected calving date until 6 weeks postpartum. Only cows that experienced normal parturition were retained in the experiment. Animals were balanced for parity and body condition score before random allocation to either a control group or an FPP-supplemented group (30 g/head/day; n = 12/group).

Diets were formulated to meet the nutrient requirements of transition and early-lactation dairy cows. FPP was administered daily by top-dressing onto the concentrate ration. Feed was offered twice daily, and the basal ration was formulated to provide approximately 3% of body weight as dry matter intake according to the nutritional requirements of growing heifers, transition cows, and lactating dairy cows (Table 1). All animals consumed the entire FPP supplement throughout the experimental period.

**Table 1:** Diet composition and nutrient profiles of the experimental concentrate formulations.

**Item**	**Concentrate for lactating cows**	**Concentrate for dry cows**	**Concentrate for heifers**
Proximate analysis (%)			
Moisture	7.79	7.18	8.15
Crude protein	20.18	17.10	19.87
Crude fat	8.14	14.82	3.27
Crude fiber	7.47	7.76	8.02
Ash	9.93	6.67	10.58
Calcium	1.18	0.64	1.31
Phosphorus	0.63	0.43	0.65
NDF	20.40	21.86	21.92
ADF	19.25	20.51	20.86
ADL	4.19	4.56	4.67
Nitrogen-free extract	46.49	46.47	50.12
Total digestible nutrients	75.09	85.23	69.24
Gross energy (kcal/kg)	4044.94	4498.89	3750.14
Ingredients (g/kg)			
Tapioca chips	440	505	425
Palm oil	50	140	—
Soybean meal	20	—	—
Sesame press cake	210	242	234
Instant soy milk powder	80	—	32
Sweet potato	100	80	213
Vitamin and mineral premix†	5	5	5
Salt	5	5	5
Sulfur	1	1	1
Monodicalcium phosphate	15	5	16
Limestone	10	—	13
Molasses	42	—	30
Sodium bicarbonate	5	—	5
Feed-grade urea	17	17	21

NDF = Neutral detergent fiber; ADF = Acid detergent fiber; ADL = Acid detergent lignin.

†Supplied per kilogram of diet: Vitamin A = 2,000,000 IU; Vitamin D₃ = 400,000 IU; Vitamin E = 3,000 IU; Vitamin K₃ = 460 mg; Vitamin B₂ = 8.8 mg; Vitamin B₁₂ = 2 mg; Cu = 400 mg; Fe = 1,000 mg; Mn = 740 mg; Co = 40 mg; Zn = 1,500 mg; I = 40 mg; Se = 4 mg; Mg = 2,640 mg.

### Sample collection

**Experiment 1:** Approximately 5 mL of blood was collected from the jugular vein into plain vacuum tubes on days 0, 15, 30, and 45 before the morning feeding (07:00-08:00 h) to minimize postprandial variation. Samples were allowed to clot and centrifuged at 3,000 × g for 15 min within 1 h of collection to obtain serum, which was stored at −80°C until analysis. Serum IGF-1 concentrations were measured after approximately 2 weeks of storage. Body weight was recorded at 15-day intervals throughout the experiment.

On day 45, skeletal muscle biopsy samples were obtained aseptically from the semitendinosus muscle using sterile Tru-cut biopsy needles (14-gauge, 15 cm; TSK Laboratory, Tochigi, Japan) following local infiltration with 2% lidocaine (Lignospan®; Septodont, Saint-Maur-des-Fossés, France). Approximately 50 mg of tissue was collected, immediately snap-frozen in liquid nitrogen, and stored at −80°C until molecular analysis.

Following biopsy collection, gentle pressure was applied to achieve hemostasis; the biopsy site was disinfected with povidone-iodine; a topical antibiotic spray was applied; and animals were monitored daily for wound healing, locomotion, appetite, and signs of inflammation or discomfort. No biopsy-related complications were observed.

**Experiment 2:** Estrous activity was monitored continuously using an automated pedometer system (Afifarm®; Afimilk Ltd., Kibbutz Afikim, Israel). Estrous alerts generated from increased locomotor activity were confirmed by transrectal ultrasonography through identification of a dominant follicle and the absence of a corpus luteum, thereby minimizing false-positive estrus detection under tropical production conditions.

All experimental procedures, sample collection, laboratory analyses, and estrus confirmation were performed by the same trained investigators in accordance with standardized operating procedures to minimize operator-related variation.

### Determination of serum IGF-1

Serum IGF-1 concentrations were measured using a bovine-specific enzyme-linked immunosorbent assay kit (CUSABIO Technology LLC, Houston, TX, USA; Catalog No. CSB-E08893b) according to the manufacturer's instructions. The assay sensitivity was 0.94 ng/mL with a detection range of 0.75-60 ng/mL. All samples were analyzed in duplicate. The intra- and inter-assay coefficients of variation were <15%. Concentrations were calculated from a seven-point standard curve generated using four-parameter logistic regression. Manufacturer validation data demonstrated acceptable recovery and assay parallelism for bovine serum.

### RNA extraction and *IGF-1* gene expression analysis

Total RNA was extracted from skeletal muscle using TRIzol™ Reagent (Thermo Fisher Scientific, Waltham, MA, USA) followed by purification with the Direct-zol RNA MiniPrep Kit (Zymo Research, Irvine, CA, USA). RNA concentration and purity were assessed using a NanoDrop spectrophotometer (Thermo Fisher Scientific), and only RNA samples with A260/A280 ratios between 1.9 and 2.1 were used.

Complementary DNA was synthesized using a commercial reverse-transcription kit according to the manufacturer's protocol. Quantitative real-time polymerase chain reaction (PCR) was performed using a CFX96 Real-Time PCR Detection System (Bio-Rad Laboratories, Hercules, CA, USA) with SYBR Green chemistry and the primers listed in Table 2. The amplification protocol consisted of initial denaturation at 95°C for 3 min followed by 40 cycles of 95°C for 10 s and 60°C for 30 s. Melt-curve analysis confirmed amplification specificity. No-template controls were included in every run to verify the absence of contamination and genomic DNA amplification.

Relative *IGF-1* gene expression was normalized against the reference gene beta-actin (*ACTB*) and calculated using the 2^−ΔΔCq^ method. All reactions were performed in triplicate. Amplification efficiencies for both target and reference genes ranged from 90% to 110%, with correlation coefficients (R²) >0.90. Single-peak melt curves confirmed amplicon specificity, and *ACTB* expression remained stable across all experimental groups.

**Table 2:** Primer sequences used for quantitative real-time polymerase chain reaction analysis.

**Gene**	**Primer**	**Sequence (5′→3′)**	**GenBank accession No.**	**Amplicon size (bp)**	**Annealing temperature (°C)**	**Reference**
IGF-1	Forward	AGTTGGTGGATGCTCTCCAGT	NM_001077828	115	60	[44]
	Reverse	CACTCATCCACGATTCCTGTC				
ACTB	Forward	GGCCGCACCACTGGCATTGTCAT	NM_173979.3	104	60	[45]
	Reverse	AGGTCCAGACGCAGGATGGCG				

*ACTB* = β-actin; bp = Base pairs; GenBank = National Center for Biotechnology Information genetic sequence database; *IGF-1* = Insulin-like growth factor-1; PCR = Polymerase chain reaction.

### Statistical analysis

Statistical analyses were performed using GraphPad Prism version 8 (GraphPad Software, San Diego, CA, USA). Data normality was evaluated using the Shapiro-Wilk test, whereas homogeneity of variance was assessed using Levene's test.

Serum IGF-1 concentrations were analyzed using two-way repeated-measures analysis of variance with treatment, time, and their interaction included as fixed effects. When significant treatment × time interactions were detected, Bonferroni-adjusted post hoc comparisons were performed.

Differences in skeletal muscle *IGF-1* gene expression and the interval from calving to first postpartum estrus were analyzed using independent-samples *t*-tests. Results are presented as mean ± SEM. Statistical significance was established at p < 0.05. Effect sizes were expressed as Cohen's *d*, and 95% confidence intervals were reported for the principal comparisons.

Because serum IGF-1 concentrations were not normally distributed at the later sampling points (Shapiro-Wilk test, p < 0.05), between-group comparisons at each sampling day were additionally evaluated using the Mann-Whitney *U* test as a complementary non-parametric analysis.

To evaluate whether the nonsignificant difference in the calving-to-first postpartum estrus interval was due to insufficient sample size, a post hoc power analysis was performed. Based on the observed effect size (Cohen's *d* = 0.80), 12 cows per group, and α = 0.05, the achieved statistical power was approximately 46%, indicating that approximately 26 cows per group would be required to achieve 80% statistical power for detecting a difference of similar magnitude.

## RESULTS

### Animal health and production performance

Throughout both experimental periods, no adverse health events attributable to FPP supplementation were observed. All animals remained clinically healthy throughout their respective study periods. In Experiment 1, mean body weight at the end of the 45-day supplementation period did not differ significantly between the control and FPP groups (312 ± 8.00 kg vs. 315 ± 9.91 kg, respectively; p > 0.05), indicating that FPP supplementation had no adverse effect on growth performance or general health status during the study.

### Effects of FPP supplementation on skeletal muscle *IGF-1* mRNA expression

**Figure 1:**
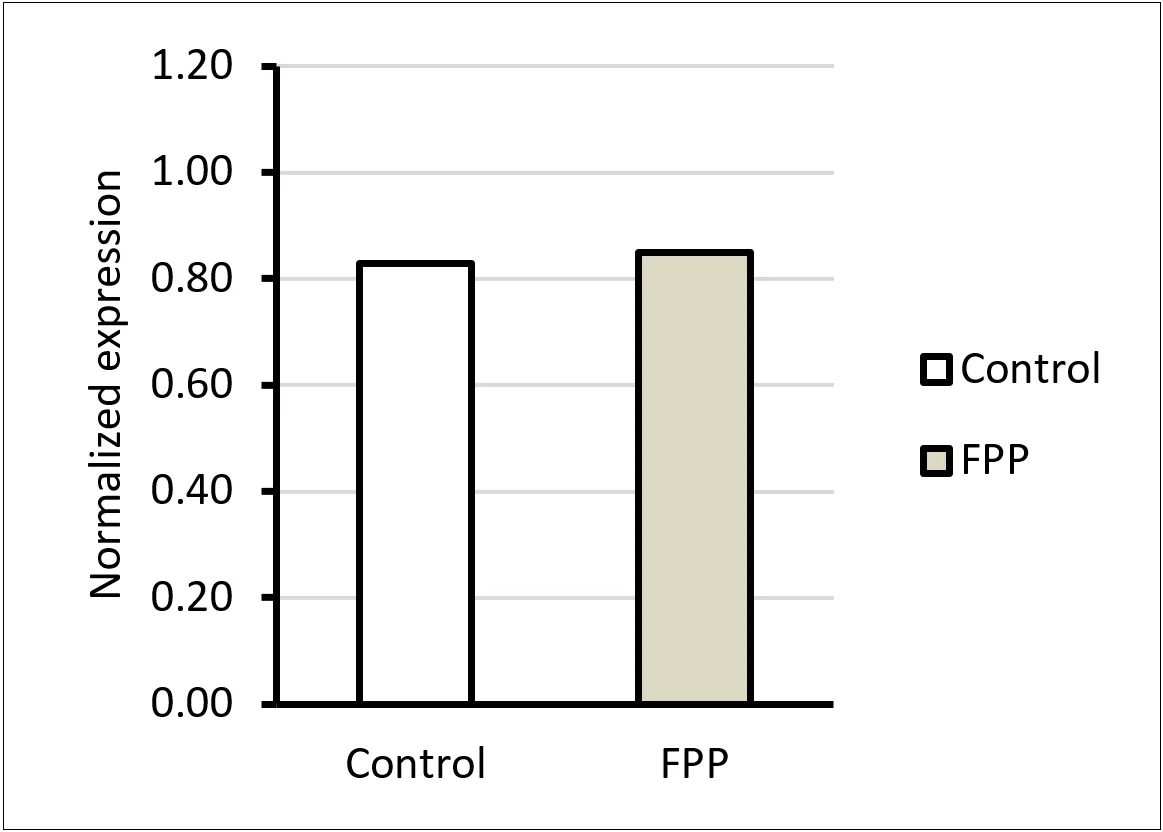
Relative skeletal muscle *IGF-1* mRNA expression in the semitendinosus muscle of the control and fermented potato protein (FPP) groups on Day 45. Data are presented as mean ± SEM (n = 5/group). No significant difference was observed between groups (p = 0.90).

Relative skeletal muscle *IGF-1* mRNA expression measured on day 45 is presented in Figure 1.

Quantitative real-time PCR analysis normalized to the reference gene *ACTB* demonstrated no significant difference in skeletal muscle *IGF-1* mRNA expression between the control and FPP groups (0.82 ± 0.07 vs. 0.84 ± 0.14, respectively; p = 0.90).

Amplification efficiencies for both the target and reference genes were within the acceptable range (90%-110%), and melt-curve analysis confirmed the presence of a single specific amplification product. Triplicate reactions demonstrated minimal technical variation, confirming the reliability and reproducibility of the gene expression analysis.

### Effects of FPP supplementation on serum IGF-1 concentrations

**Figure 2:**
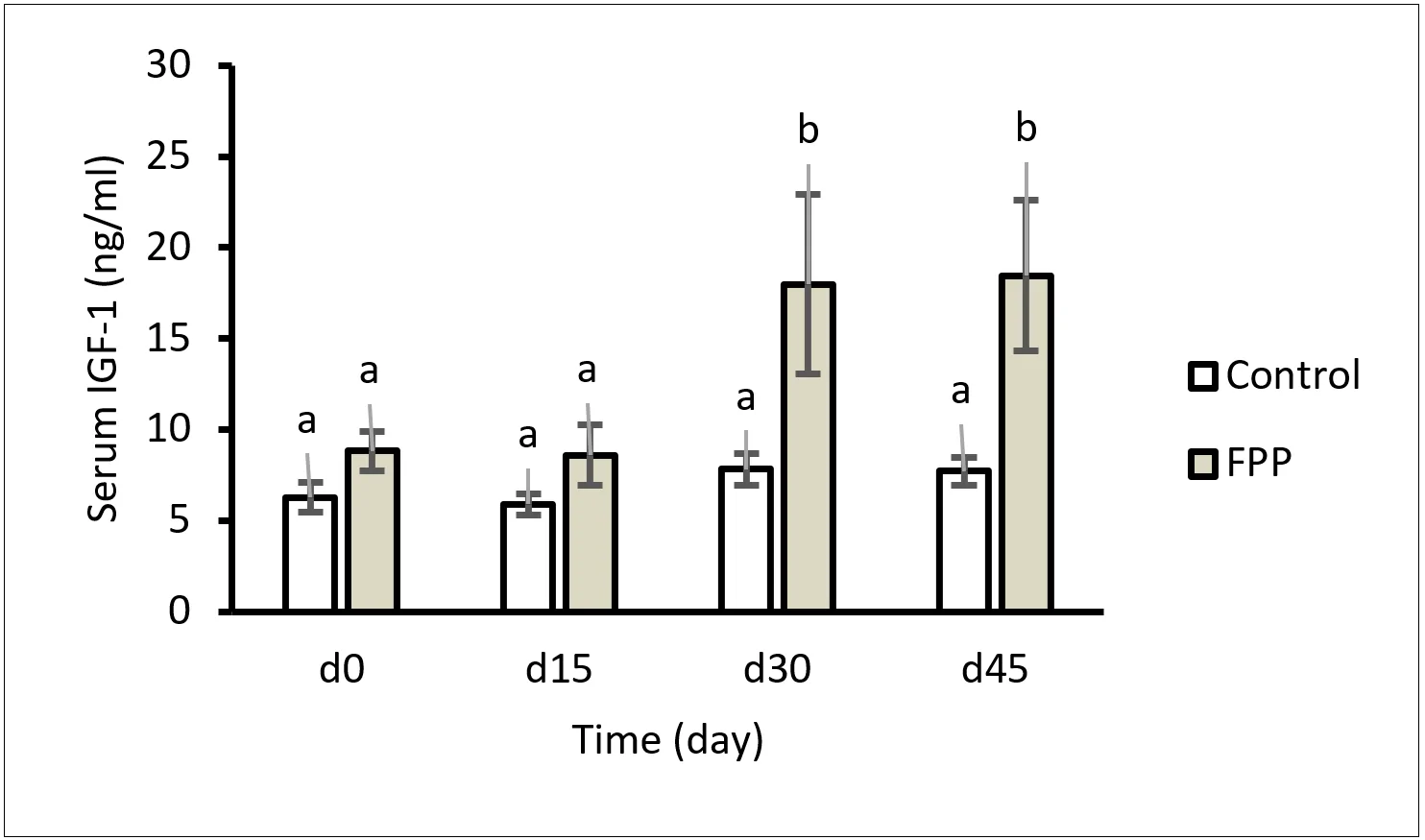
Serum IGF-1 concentrations in the control and fermented potato protein (FPP) groups during the 45-day supplementation period. Data are presented as mean ± SEM (n = 5/group). Different lowercase letters indicate significant differences between groups within the same sampling day (p < 0.05).

Changes in serum IGF-1 concentrations during the supplementation period are presented in Figure 2. Serum IGF-1 concentrations did not differ significantly between the control and FPP groups at baseline (day 0; p = 0.056) or after 15 days of supplementation (day 15; p = 0.151; Mann-Whitney *U* test).

However, significant differences were observed during the later stages of supplementation. On day 30, cows receiving FPP supplementation exhibited significantly higher serum IGF-1 concentrations than those in the control group (17.97 ± 4.93 ng/mL vs. 7.82 ± 0.87 ng/mL, respectively; p = 0.016). This difference persisted on day 45, with the FPP group maintaining significantly higher circulating IGF-1 concentrations than the control group (18.45 ± 4.13 ng/mL vs. 7.72 ± 0.75 ng/mL; p = 0.016).

The observed between-group differences corresponded to large effect sizes, with Cohen's *d* values of 1.28 and 1.61 on days 30 and 45, respectively.

Within-group analysis demonstrated a progressive increase in circulating IGF-1 concentrations throughout the supplementation period in the FPP group, whereas concentrations remained relatively stable in the control group.

### Effects of FPP supplementation on postpartum reproductive recovery

**Figure 3:**
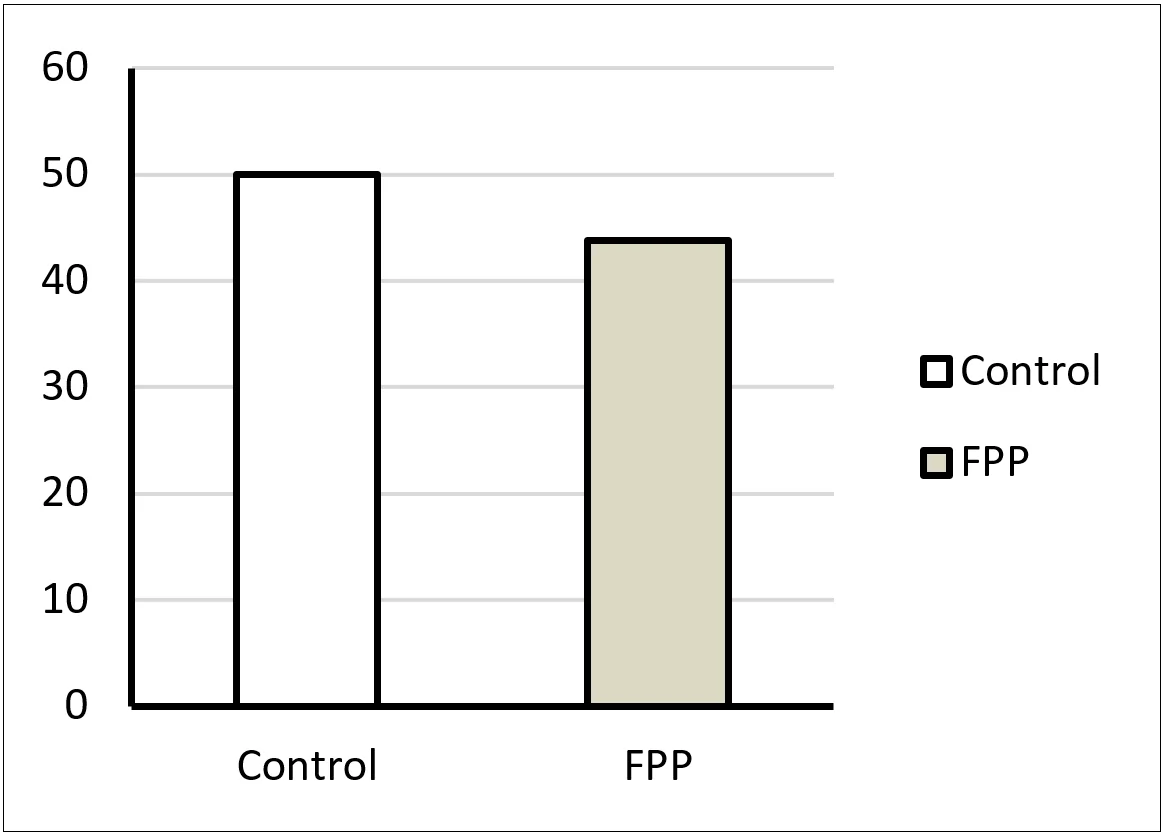
Interval from calving to first postpartum estrus in the control and fermented potato protein (FPP) groups. Data are presented as mean ± SEM (n = 12/group). No significant difference was observed between groups (p = 0.06).

The interval from calving to first postpartum estrus is presented in Figure 3.

All 24 enrolled dairy cows completed the reproductive monitoring period from 3 weeks prepartum to 6 weeks postpartum. No severe postpartum disorders were observed during the study, and body condition scores remained within the predetermined range (2.75-3.25) for all animals.

Cows receiving FPP supplementation exhibited a numerically shorter interval from calving to first postpartum estrus than control cows (43.83 ± 2.40 days vs. 50.00 ± 2.06 days, respectively). However, the difference did not reach statistical significance (p = 0.06). The estimated mean difference between groups was 6.17 days (95% confidence interval: −0.39 to 12.73 days), corresponding to a large effect size (Cohen's *d* = 0.80).

Although statistical significance was not achieved, the magnitude of the observed effect suggests a biologically relevant improvement in postpartum reproductive recovery following FPP supplementation.

## DISCUSSION

### FPP enhances systemic endocrine regulation through the somatotropic axis

The present study demonstrated that dietary FPP supplementation increased circulating IGF-1 concentrations in dairy cattle, whereas no significant changes were observed in skeletal muscle *IGF-1* mRNA expression or the interval from calving to first postpartum estrus. These findings indicate that FPP primarily modulates systemic endocrine regulation rather than local tissue-specific gene expression and may contribute to metabolic adaptations associated with postpartum reproductive recovery.

In ruminants, circulating IGF-1 is synthesized predominantly in the liver in response to growth hormone (GH) stimulation and represents the principal endocrine component of the somatotropic axis [16, 17]. During early lactation, dairy cows commonly experience hepatic GH resistance characterized by reduced GH receptor (*GHR*) expression and impaired downstream signaling despite elevated circulating GH concentrations [10, 15, 17]. This uncoupling of the GH-IGF-1 axis is closely associated with NEB, reduced hepatic IGF-1 synthesis, and delayed resumption of ovarian cyclicity [2, 15].

The progressive increase in circulating IGF-1 observed after approximately 30 days of supplementation suggests gradual restoration of hepatic endocrine function rather than an immediate metabolic response. Fermentation improves protein digestibility, increases the availability of essential amino acids, and generates bioactive peptides that may enhance hepatic metabolism and endocrine signaling [36–38, 40]. Adequate amino acid availability has been reported to promote *GHR* expression and activate the Janus kinase 2 (*JAK2*)-signal transducer and activator of transcription 5 (*STAT5*) signaling pathway, which regulates hepatic *IGF-1* transcription [17, 36–38]. Improved nutritional status may also increase circulating insulin concentrations, facilitating recoupling of the somatotropic axis [11, 17, 36]. Nevertheless, direct evidence that FPP-derived metabolites, including indole-3-acetic acid, activate hepatic GH signaling or the *JAK2/STAT5* pathway in dairy cattle is currently unavailable [40, 41, 43, 45]. Therefore, the present findings are more appropriately interpreted as evidence of improved nutritional status rather than direct endocrine stimulation by FPP, and the precise molecular mechanisms remain to be established [17, 26–28].

Beyond hepatic synthesis, the biological activity of circulating IGF-1 is regulated by IGFBPs, particularly IGFBP-2 and IGFBP-3 [20, 28]. IGFBP-3 stabilizes circulating IGF-1 by forming a ternary complex with the acid-labile subunit, whereas IGFBP-2 is generally increased during catabolic conditions and reduces IGF-1 bioavailability [20, 26–28]. Although IGFBPs were not measured in the present study, the increased circulating IGF-1 observed after FPP supplementation may reflect both enhanced hepatic synthesis and a more favorable IGFBP profile, thereby improving IGF-1 stability and endocrine activity.

The delayed endocrine response observed in this study is consistent with previous reports demonstrating that improvements in the somatotropic axis occur gradually as animals transition from a catabolic to a more anabolic state [17, 36, 37]. Moreover, the evaluated supplementation level (30 g/head/day) falls within the biologically effective range previously reported for fermented concentrated potato protein (25-50 g/head/day) [41], indicating that commercially practical inclusion rates can improve endocrine status under tropical production conditions. However, because only a single supplementation level was evaluated, future dose-response studies are warranted.

### Tissue-specific regulation of skeletal muscle *IGF-1*

Despite the increase in circulating IGF-1, skeletal muscle *IGF-1* mRNA expression remained unchanged following FPP supplementation. This finding is consistent with previous studies demonstrating that circulating IGF-1 is regulated primarily by hepatic synthesis, whereas skeletal muscle *IGF-1* acts predominantly via autocrine and paracrine mechanisms that control muscle growth and protein turnover [16, 18–20]. Consequently, the absence of changes in skeletal muscle *IGF-1* expression does not contradict the endocrine response; rather, it highlights tissue-specific regulation of the IGF system.

Furthermore, mRNA abundance does not necessarily reflect protein abundance or biological activity, as post-transcriptional regulation, translational efficiency, and protein turnover can independently influence local signaling [18–20]. Therefore, future investigations should quantify skeletal muscle IGF-1 protein concentrations, along with downstream signaling molecules such as phosphorylated AKT and the mammalian target of rapamycin, to clarify local biological responses.

Consistent with previous studies in Holstein cows supplemented with fermented concentrated potato protein [41], the present study demonstrated a delayed increase in circulating IGF-1 after approximately 30 days of supplementation. However, to our knowledge, this is the first study to simultaneously evaluate systemic endocrine responses and skeletal muscle *IGF-1* gene expression in tropical crossbred dairy cattle, providing novel evidence that FPP primarily influences systemic endocrine regulation rather than local *IGF-1* expression in skeletal muscle under tropical production conditions.

### Implications for postpartum reproductive recovery

Although FPP supplementation did not significantly shorten the interval from calving to first postpartum estrus, supplemented cows exhibited a biologically meaningful numerical reduction of approximately 6 days. Even modest reductions in the postpartum anestrous interval can improve reproductive efficiency and economic performance in dairy herds [2, 5, 6]. The concurrent increase in circulating IGF-1 together with the shorter postpartum estrus interval is consistent with the established role of IGF-1 in promoting granulosa cell proliferation, estradiol synthesis, follicular responsiveness to gonadotropins, follicular development, and ovulation [23–25]. IGF-1 may also influence reproductive function centrally by stimulating hypothalamic kisspeptin neurons and activating the HPG axis [33–35].

Because endocrine measurements and reproductive outcomes were obtained from separate experimental cohorts, a direct correlation between circulating IGF-1 concentration and reproductive performance could not be established. Nevertheless, the present findings suggest that elevated circulating IGF-1 can occur independently of skeletal muscle *IGF-1* expression, supporting a predominantly systemic mechanism of action [16, 18–20, 38].

No adverse effects on voluntary feed intake or milk production were observed, indicating that supplementation with 30 g/head/day was well tolerated under the experimental conditions, although these parameters were recorded descriptively and were not statistically analyzed. Furthermore, as a value-added agro-industrial by-product, FPP may represent a sustainable nutritional strategy that reduces dependence on imported protein sources while improving metabolic adaptation in tropical dairy production systems where feed quality is frequently variable [40].

### Strengths, limitations, and future perspectives

A major strength of this study is the integrated evaluation of endocrine, molecular, and reproductive responses to FPP supplementation, providing novel mechanistic insight into the regulation of the somatotropic axis in tropical crossbred dairy cattle.

Nevertheless, several limitations should be acknowledged. The study was conducted under tropical field conditions, where seasonal variation, heat stress, and other environmental factors could not be completely controlled. The relatively small sample size in Experiment 1 may have reduced statistical power to detect subtle changes in skeletal muscle *IGF-1* expression. In addition, important components of the somatotropic axis, including hepatic *GHR* expression, circulating insulin, IGFBPs, non-esterified fatty acids, β-hydroxybutyrate, glucose, and inflammatory biomarkers, were not measured. Feed intake and milk production were monitored but were not predefined study outcomes and therefore were not statistically analyzed.

Reproductive performance was evaluated only until first postpartum estrus, whereas longer-term fertility indicators, including conception rate at first service, pregnancy rate, services per conception, days open, and calving interval, were not investigated. Furthermore, endocrine measurements and reproductive outcomes were obtained from separate cohorts, preventing direct within-animal association analyses. Finally, a post hoc sensitivity analysis indicated that the reproductive experiment had approximately 46% statistical power, suggesting that approximately 26 cows per treatment group would be required to detect the observed effect size with 80% power.

Future studies should include larger, multi-herd populations, evaluate multiple supplementation levels, integrate endocrine and reproductive measurements in the same animals, quantify hepatic signaling pathways and metabolic biomarkers, determine long-term reproductive performance, and assess the economic feasibility of FPP supplementation in commercial tropical dairy production systems.

## CONCLUSION

Dietary supplementation with FPP at 30 g/head/day significantly increased circulating IGF-1 concentrations after 30 days of supplementation, whereas skeletal muscle *IGF-1* mRNA expression remained unchanged. In addition, FPP supplementation produced a numerically shorter interval from calving to first postpartum estrus, although this difference was not statistically significant. These findings indicate that FPP primarily modulates systemic endocrine regulation through the somatotropic axis rather than local skeletal muscle *IGF-1* expression and may support metabolic adaptations associated with postpartum reproductive recovery.

From a practical perspective, FPP represents a promising functional feed ingredient derived from an agro-industrial by-product that can be incorporated into transition dairy cow diets without adverse effects on animal health. Its ability to increase circulating IGF-1 at a commercially relevant supplementation level suggests potential value for improving metabolic resilience during the transition period, particularly in tropical dairy production systems where heat stress and variable feed quality frequently compromise reproductive performance. The use of FPP may therefore provide a sustainable nutritional strategy to support endocrine function while adding value to agricultural by-products.

Overall, this study provides the first integrated evidence that FPP supplementation enhances systemic endocrine responses without altering *IGF-1* gene expression in skeletal muscle in tropical crossbred dairy cattle. Although additional studies with larger populations, multiple supplementation levels, comprehensive metabolic profiling, and long-term fertility assessments are warranted, the present findings establish a scientific basis for further investigation of FPP as a nutritional approach to improve metabolic adaptation and reproductive efficiency in dairy cattle under tropical production conditions.

## DATA AVAILABILITY

The data generated during the study are included in the manuscript.

## GENERATIVE AI DECLARATION

The authors declare that generative artificial intelligence (AI) tools were used solely to improve language, grammar, and readability during manuscript preparation. All scientific content, data analysis, interpretation of results, and conclusions were developed and verified by the authors. The authors take full responsibility for the accuracy, integrity, and originality of the work presented, and no AI tool was listed as an author.

## AUTHORS’ CONTRIBUTIONS

SS: Conceptualization, formal analysis, funding acquisition, investigation, methodology, writing–original draft, and writing–review and editing. SK, PI, and SA: Investigation and methodology. TS: Formal analysis, investigation, methodology, and writing–review and editing. NT: Conceptualization, supervision, and writing–review and editing. All authors have read and approved the final manuscript.

## References

[1] Walsh SW, Williams EJ, Evans ACO (2020). A review of the causes of poor fertility in high milk producing dairy cows. Anim Reprod Sci.

[2] Garnsworthy PC, Sinclair KD, Webb R (2021). Integration of physiological mechanisms that influence fertility in dairy cows. J Dairy Sci.

[3] Sammad A, Khan MZ, Abbas Z, Hu L, Ullah Q, Wang Y, Zhu H, Wang Y (2022). Major nutritional metabolic alterations influencing the reproductive system of postpartum dairy cows. Metabolites.

[4] Lucy MC, Butler ST, Garverick HA (2021). Endocrine and metabolic mechanisms linking nutrition and reproduction in postpartum dairy cows. J Dairy Sci.

[5] Diskin MG, Kenny DA (2021). Optimising reproductive performance in cattle. Theriogenology.

[6] Lopez-Gatius F (2024). Advances in dairy cattle reproduction in the last decade. Animals.

[7] Opsomer G, Mijten P, Coryn M, de Kruif A (1996). Post-partum anoestrus in dairy cows: a review. Vet Q.

[8] Rhodes FM, McDougall S, Burke CR, Verkerk GA, Macmillan KL (2003). Invited review: Treatment of cows with an extended postpartum anestrous interval. J Dairy Sci.

[9] Triwutanon S, Rukkwamsuk T (2023). Factors affecting first ovulation in postpartum dairy cows under tropical conditions: a review. Open Vet J.

[10] Roche JR, Burke CR, Crookenden MA, Heiser A, Loor JL, Meier S, Mitchell MD, Phyn CVC, Turner S-A (2022). Fertility and the transition dairy cow. J Dairy Sci.

[11] Leroy JLMR, Vanholder T, Delanghe JR, Opsomer G, Van Soom A (2020). Metabolism and fertility in dairy cows. Reproduction.

[12] Mohapatra SS, Mukherjee J, Banerjee D, Das PK, Ghosh PR, Das K (2021). RFamide peptides, the novel regulators of mammalian HPG axis: a review. Vet World.

[13] Wiltbank MC, Souza AH, Carvalho PD (2022). Hormonal regulation of postpartum ovarian function in dairy cows. Theriogenology.

[14] Wiltbank MC, Baez GM, Garcia-Guerra A, Toledo MZ, Monteiro PLJ, Melo LF (2020). Physiology of the bovine estrous cycle. Theriogenology.

[15] Butler WR (2021). Negative energy balance and postpartum fertility in dairy cows. Theriogenology.

[16] Sjögren K, Liu JL, Blad K, Skrtic S, Vidal O, Wallenius V, LeRoith D, Törnell J, Isaksson OG, Jansson JO, Ohlsson C (1999). Liver-derived IGF-I is the principal source of circulating IGF-I. Proc Natl Acad Sci USA.

[17] Lucy MC (2021). The somatotropic axis and dairy cow fertility. J Dairy Sci.

[18] Ghanipoor-Samami M, Javadmanesh A, Burns BM, Thomsen DA, Nattrass GS, Estrella CAS, Kind KL, Hiendleder S (2018). Atlas of tissue- and developmental stage-specific gene expression for the bovine insulin-like growth factor system. PLoS One.

[19] Meiyu Q, Roth Z, Di L (2011). Insulin-like growth factor-I in the reproductive system of female bovine. J Northeast Agric Univ.

[20] Neirijnck Y, Papaioannou MD, Nef S (2019). The insulin/IGF system in mammalian sexual development and reproduction. Int J Mol Sci.

[21] Collier RJ, Bauman DE, Baumgard LH (2025). Somatotropin and lactation biology. J Dairy Sci.

[22] Saleh AA, Hassan TGM, El-Hedainy DKA, El-Barbary ASA, Sharaby MA, Hafez EE, Rashad AMA (2024). IGF-I and GH genes polymorphism and their association with milk yields, composition and reproductive performance in Holstein–Friesian dairy cattle. BMC Vet Res.

[23] Bisinotto RS, Ribeiro ES, Lima FS, Martinez N, Greco LF, Barbosa LF (2021). Role of metabolic hormones including IGF-1 in follicular development in dairy cattle. Theriogenology.

[24] Wiltbank MC, Souza AH, Carvalho PD, Cunha AP, Giordano JO, Fricke PM (2022). Regulation of follicle selection and ovulation in cattle. Anim Reprod.

[25] Butler ST, Roche JF (2020). Metabolic regulation of follicular development in dairy cows. Domest Anim Endocrinol.

[26] Santos JEP, Ribeiro ES, Bisinotto RS, Lima FS, Greco LF (2021). Nutrition and reproduction in dairy cattle. Anim Reprod.

[27] Bisinotto RS, Ribeiro ES, Lima FS, Martinez N, Greco LF, Santos JEP (2021). Metabolic indicators of fertility in dairy cattle. J Dairy Sci.

[28] Schiffers C, Serbetci I, Mense K, Kassens A, Grothmann H, Sommer M, Hoeflich C, Hoeflich A, Bollwein H, Schmicke M (2024). Association between IGF-1 and IGFBPs in blood and follicular fluid in dairy cows under field conditions. Animals.

[29] d’Anglemont de Tassigny X, Colledge WH (2010). The role of kisspeptin signaling in reproduction. Physiology.

[30] Uenoyama Y, Nagae M, Tsuchida H, Inoue N, Tsukamura H (2021). Role of KNDy neurons as GnRH pulse generator. Front Endocrinol.

[31] Harter CJ, Kavanagh GS, Smith JT (2018). The role of kisspeptin neurons in reproduction and metabolism. J Endocrinol.

[32] Xie Q, Kang Y, Zhang C, Xie Y, Wang C, Liu J, Yu C, Zhao H, Huang D (2022). Kisspeptin in control of the hypothalamic–pituitary–gonadal axis. Front Endocrinol.

[33] Neal-Perry G, Yao D, Shu J, Sun Y, Etgen AM (2014). Insulin-like growth factor-I regulates LH release by modulation of kisspeptin and NMDA-mediated neurotransmission. Endocrinology.

[34] Hiney JK, Srivastava VK, Anderson DNV, Hartzoge NL, Dees WL (2018). Regulation of kisspeptin synthesis by insulin-like growth factor-1. Alcohol Clin Exp Res.

[35] Dees WL, Hiney JK, Srivastava VK (2021). Insulin-like growth factor-1 influences gonadotropin-releasing hormone regulation of puberty. Neuroendocrinology.

[36] Gilbreath KR, Bazer FW, Satterfield MC, Wu G (2021). Amino acid nutrition and reproductive performance in ruminants. Adv Exp Med Biol.

[37] Zhang M, Xu J, Wang T, Wan X, Zhang F, Wang L, Zhu X, Gao P, Shu G, Jiang Q, Wang S (2018). The dipeptide Pro-Gly promotes IGF-1 expression and secretion in HepG2 and female mice via PepT1-JAK2/STAT5 pathway. Front Endocrinol (Lausanne).

[38] Wan X, Wang S, Xu J, Zhuang L, Xing K, Zhang M, Zhu X, Wang L, Gao P, Xi Q, Sun J, Zhang Y, Li T, Shu G, Jiang Q (2017). Dietary protein-induced hepatic IGF-1 secretion mediated by PPARγ activation. PLoS One.

[39] Desta AG (2024). The effect of crude protein and energy on conception of dairy cows: a review. Discov Anim.

[40] Missotten JAM, Michiels J, Degroote J, De Smet S (2015). Fermented liquid feed for pigs: an ancient technique for the future. J Anim Sci Biotechnol.

[41] Aktuğ E, Baytok E, Türkmenoğlu B (2023). Effect of fermented concentrated potato protein on milk yield and fertility parameters in dairy cows in the prepartum and postpartum periods. Large Anim Rev.

[42] Krasaesub S, Sukmak M, Intaravichai P, Boodde O, Thanantong N, Sajapitak S (2022). Effects of feed restriction and fermented potato protein supplementation on IGF1, GHR and Kiss1 mRNA expression in intact nursery gilts.

[43] Xin Q, Uyanga VA, Jiao H, Zhao J, Wang X, Li H, Zhou Y, Lin H (2022). Insulin-like growth factor-1 is involved in the deteriorated performance of aged laying hens. J Anim Sci.

[44] Kelly GM, Buckley DA, Kiely PA, Adams DR, O’Connor R (2012). Serine phosphorylation of the insulin-like growth factor-I (IGF-1) receptor C-terminal tail restrains kinase activity and cell growth. J Biol Chem.

[45] Zhuo Y, Zhou D, Che L, Fang Z, Lin Y, Wu D (2014). Feeding prepubescent gilts a high-fat diet induces molecular changes in the hypothalamus-pituitary-gonadal axis and predicts early timing of puberty. Nutrition.

